# Sex initiates adaptive evolution by recombination between beneficial loci

**DOI:** 10.1371/journal.pone.0177895

**Published:** 2017-06-02

**Authors:** Thomas Scheuerl, Claus-Peter Stelzer

**Affiliations:** 1 Research Institute for Limnology, University of Innsbruck, Mondsee, Austria; 2 Imperial College London, Silwood Park Campus, London, United Kingdom; 3 Department of Food and Environmental Sciences/Microbiology and Biotechnology, University of Helsinki, Helsinki, Finland; Fred Hutchinson Cancer Research Center, UNITED STATES

## Abstract

Current theory proposes that sex can increase genetic variation and produce high fitness genotypes if genetic associations between alleles at different loci are non-random. In case beneficial and deleterious alleles at different loci are in linkage disequilibrium, sex may i) recombine beneficial alleles of different loci, ii) liberate beneficial alleles from genetic backgrounds of low fitness, or iii) recombine deleterious mutations for more effective elimination. In our study, we found that the first mechanism dominated the initial phase of adaptive evolution in *Brachionus calyciflorus* rotifers during a natural selection experiment. We used populations that had been locally adapted to two environments previously, creating a linkage disequilibrium between beneficial and deleterious alleles at different loci in a combined environment. We observed the highest fitness increase when several beneficial alleles of different loci could be recombined, while the other mechanisms were ineffective. Our study thus provides evidence for the hypothesis that sex can speed up adaptation by recombination between beneficial alleles of different loci, in particular during early stages of adaptive evolution in our system. We also suggest that the benefits of sex might change over time and state of adaptive progress.

## Introduction

Sex is thought to facilitate natural selection by increasing genetic variation [1–3] and the rate of fitness increase equals the additive genetic variance [4] of the population. However, only if genetic variation is in negative linkage disequilibrium (-LD) [3,5,6], when associations between alleles at different loci are non-random and intermediate fitness genotypes are in excess, sex changes variation affecting fitness. This occurs via three genetic mechanisms [7,8]: It may i) recombine beneficial alleles of different loci, ii) liberate beneficial alleles from genetic backgrounds of low fitness, or iii) recombine deleterious mutations for more effective elimination [9–15]. Please note, by non-random associations we refer to associations between alleles at different loci [16,17], rather than associations between alleles at the same locus. There is convincing theoretical evidence for the key role of -LD (produced by drift or epistasis) [18], and experiments have [19,20] demonstrated that sex may have positive long- and short-term fitness effects [16,20]. However, experimental evidence for the dominance of one of the three genetic mechanisms to initiate adaptive evolution is rare [7,8,21] and contradictory [10,12,14,22,23]. To resolve the relative importance of the mechanisms one should compare populations with deleterious and beneficial mutations adapting to new conditions [23], which we have done here using populations of the rotifer *Brachionus calyciflorus*.

*B*. *calyciflorus* rotifers are short-lived (~7–10 days), diploid, cyclical parthenogens [24] (see S1 File for details). They feed on unicellular microalgae (here *Chlamydomonas reinhardtii*) and initiate sexual reproduction at population densities higher than ~1 female ml^-1^. Previously, we locally adapted our populations to either low-food or high-salt environments (F-Environment and S-Environment respectively, see Figure 3 of [24]) to obtain F- and S-Populations [24]. These locally adapted populations were less fit in the alternative environment, so beneficial alleles for one selection regime were linked with different loci of low fitness in the alternative regime [25]. In this experiment, we used a combined environment (F-S-Environment) and selected populations consisting of either F- or S-Populations, mixtures of them (F-S-Populations) and populations consisting of the ancestral clones (A-Populations). We used four replicates per population and followed short-term fitness change (Box 1). The local adaptation of the F- and S-Populations should establish a -LD opposing directional selection in the combined environment [26]. Since populations have not previously experienced the combined environment (F-S-Environment), we assumed intermediate fitness phenotypes only. Thus, sex should have the potential to purge deleterious mutations and liberate beneficial alleles from genetic backgrounds of low fitness. Likewise, genetic variance might be reduced after local adaptation. Thus, mean population fitness was expected to change after several generations in the combined environment, either due to sex causing an increase in variation, or because of new, beneficial mutations (Box 1).

**Box 1. The theoretical prediction on mean population fitness over short-time-scales/few-generations when populations have different genetic structures**

**Fig 1:**
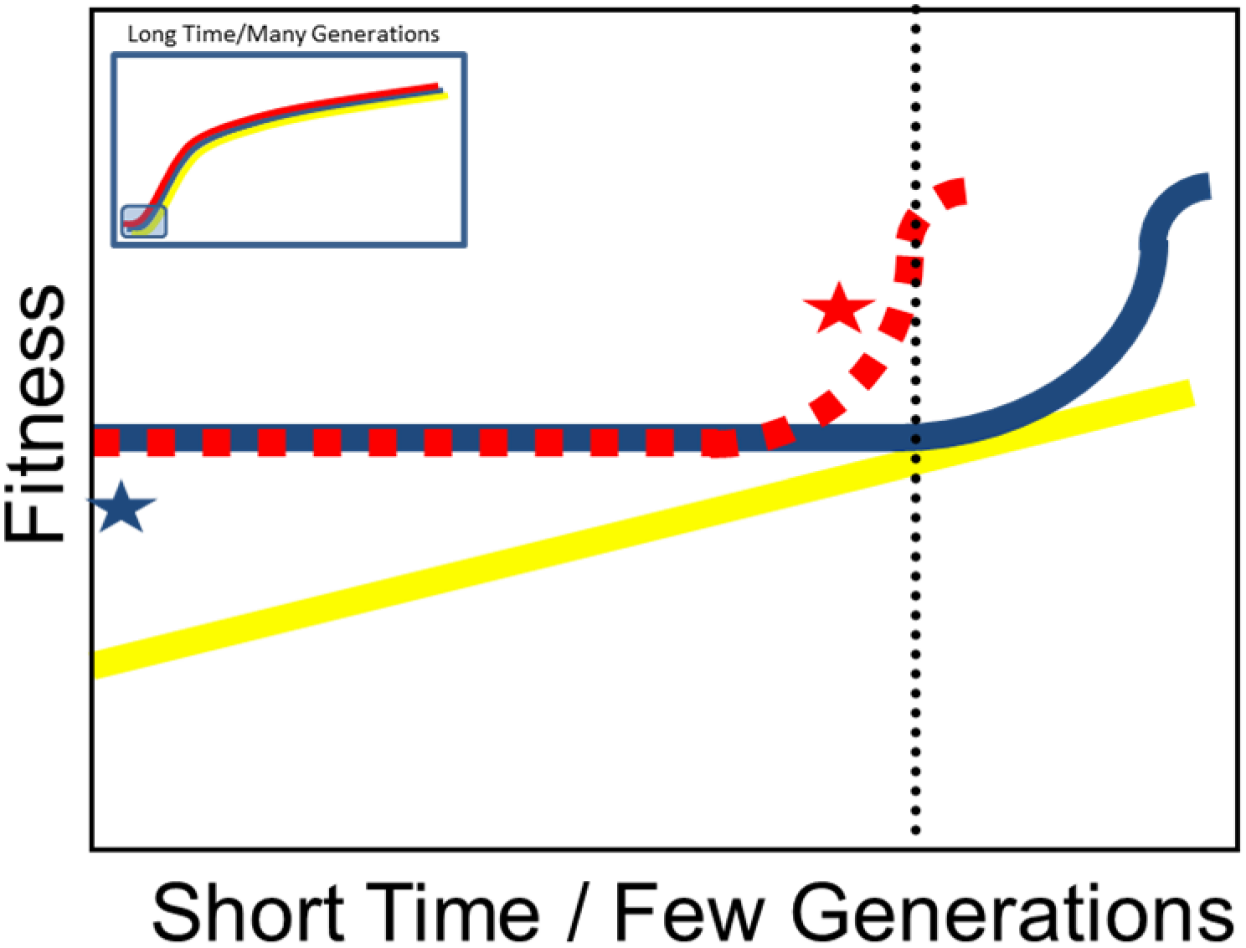

While fitness change is reported to be power-law distributed over many generations after an initial phase (Fig 1 insert), we were interested which process initiates this fitness change right after a few generations (Fig 1). In locally adapted populations with only one beneficial trait associated with a negative genetic background (blue line; S- or F-Populations), fitness will be initially high (blue star), but mean population fitness will primarily remain on a constant level due to reduced genetic variation. After some generations sex can liberate these beneficial loci from the negative associations, or purge deleterious mutations and fitness will increase. If the important mechanism for adaptation is recombining two present beneficial loci from different individuals (red broken line; F-S-Populations), there should be a change in fitness (red star) before this happens in the population with only one beneficial trait. The time point of interest is indicated by the black broken line. In populations with high genetic variation and no pre-adaptation, fitness should constantly increase, initiated by clonal erosion and loss of low fitness genotypes (yellow line).

In the F-S-Populations, beneficial alleles at different loci for both environmental conditions (F and S) are already present at the start of the experiment and can be recombined. If this is the dominant genetic mechanism, we would expect that these populations increase in fitness before a response to selection is observed in the other populations (Box 1). Finally, in the A-Populations, we expect an immediate fitness change due to clonal sorting (Box 1). We defined fitness as the mean change in absolute population growth rate day^-1^ during four-day periods between transfers to fresh conditions, which corresponds to ~2–3 asexual and ~0.5 sexual generations (see S1 File). After each transfer population density was re-set to ~2.2 females ml^-1^.

## Results

### Fitness increased if several beneficial traits could be recombined

**Fig 2:**
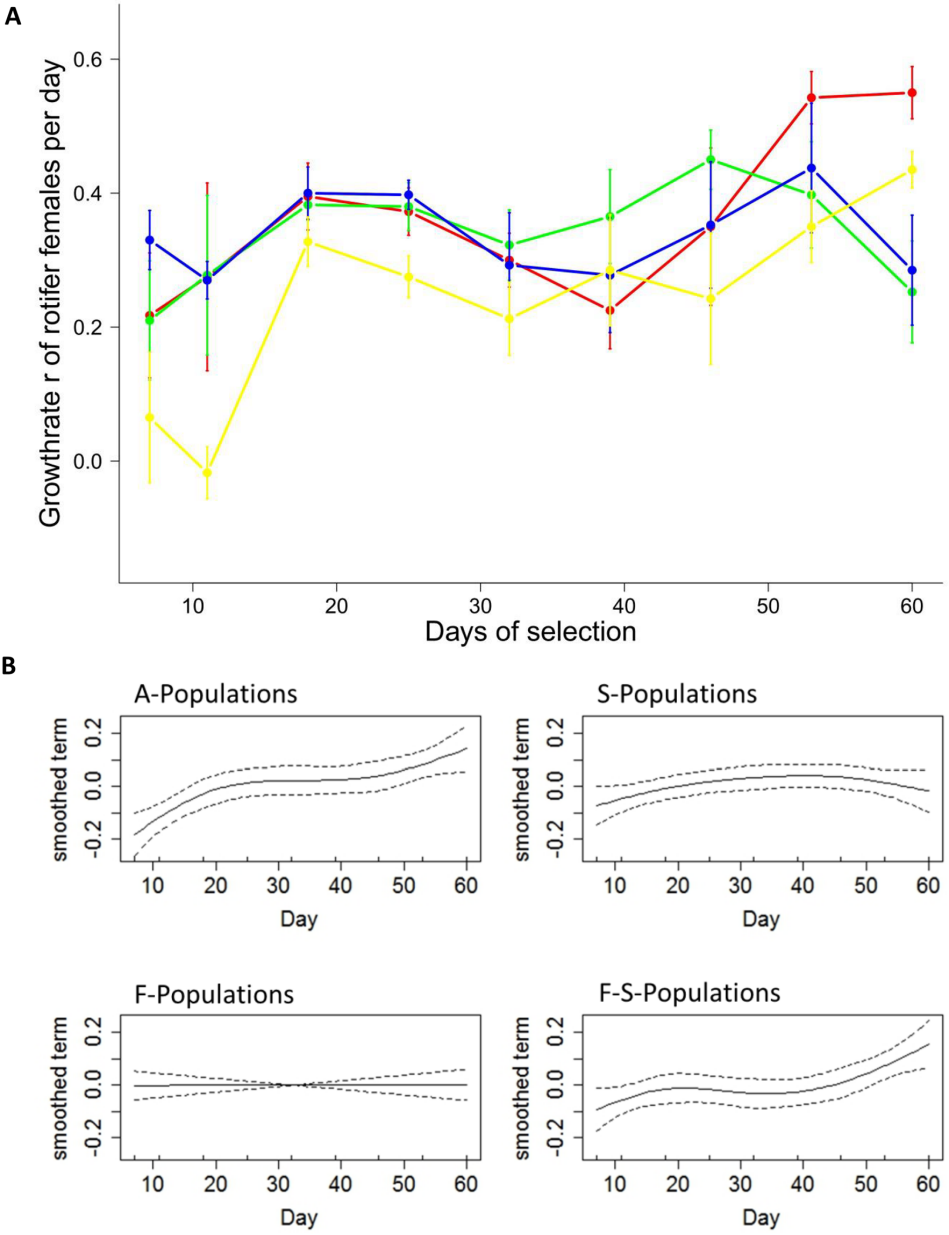
The change in absolute mean population fitness over time for *Brachionus calyciflorus* rotifers in the F-S-Environment. (A) *In situ* measure of growth rates in populations with different genetic structures (dots represent mean values, bars ±1 standard error) and (B) the smoothed term from the GAMM on fitness (dashed line shows the 95% confidence interval), both plotted over time. (A) Changes were recorded for small number of generations (~2–3 asexual and maximum 1 sexual generations). Yellow: A-Populations; Blue: F-Populations; Green: S-Populations; Red: F-S-Populations. Counts are based on three independent samples from each replicate population (n = 4).

We followed the short-term change in fitness in our populations over 60 days. This corresponds to 30 asexual and 3–4 sexual generations, as estimated from measured growth rates (~0.5 day^-1^) and by taking into account that sexual reproduction is delayed for ~5–10 asexual generations in females hatched from diapausing eggs [27,28]. In the F-S-Environment population fitness only changed in the A-Populations and F-S-Populations (Fig 2).

The initial fitness level of S-Populations was slightly higher compared to the A-Populations (GAMM-Intercept: Fig 2A, S1 Table; Estimate = 0.0958, t-value = 2.9440, P = 0.0038), which indicates the local adaptation to the high salt condition, while all other populations had an even higher fitness level. Fitness in the A-Populations constantly increased over time as indicated by the smoothed slope (GAMM-Smooth-Term: Fig 2B and S1 Table; Ref.df = 2.5320, F = 9.0380, P<0.0001). For S- and F-Populations, mean population fitness did not change over time (GAMM-Smooth-Term: Fig 2B and S1 Table. S-P: Ref.df = 2.1430, F = 2.2930, P = 0.1006; FP: Ref.df = 1, F = 0.0400, P = 0.8417). In the F-S-Populations, fitness did not change initially, but increased significantly towards the end of the experiment (GAMM-Smooth-Term: Fig 2B, S1 Table. Ref.df = 2.8970, F = 5.7840, P = 0.0012). Over the course of the experiment there were noticeable, although non-significant, fluctuations in fitness. We assume that these fluctuations were caused by experimental noise and emphasize that only in the A- and F-S-Populations we observed consistent and significant increases in fitness.

There was considerable change in mean fitness, while variation in fitness remained high over time for the F-S- and A-Populations (S1 Fig. mean = 1.24, standard deviation = 0.57, and mean = 1.56, standard deviation = 0.75, respectively). In contrast, mean and variance of fitness were lower in the F- and S-Populations (mean = 0.99, standard deviation = 0.51, and mean = 1.08 and standard deviation = 0.42).

### No differences in sexual rates across populations

**Fig 3:**
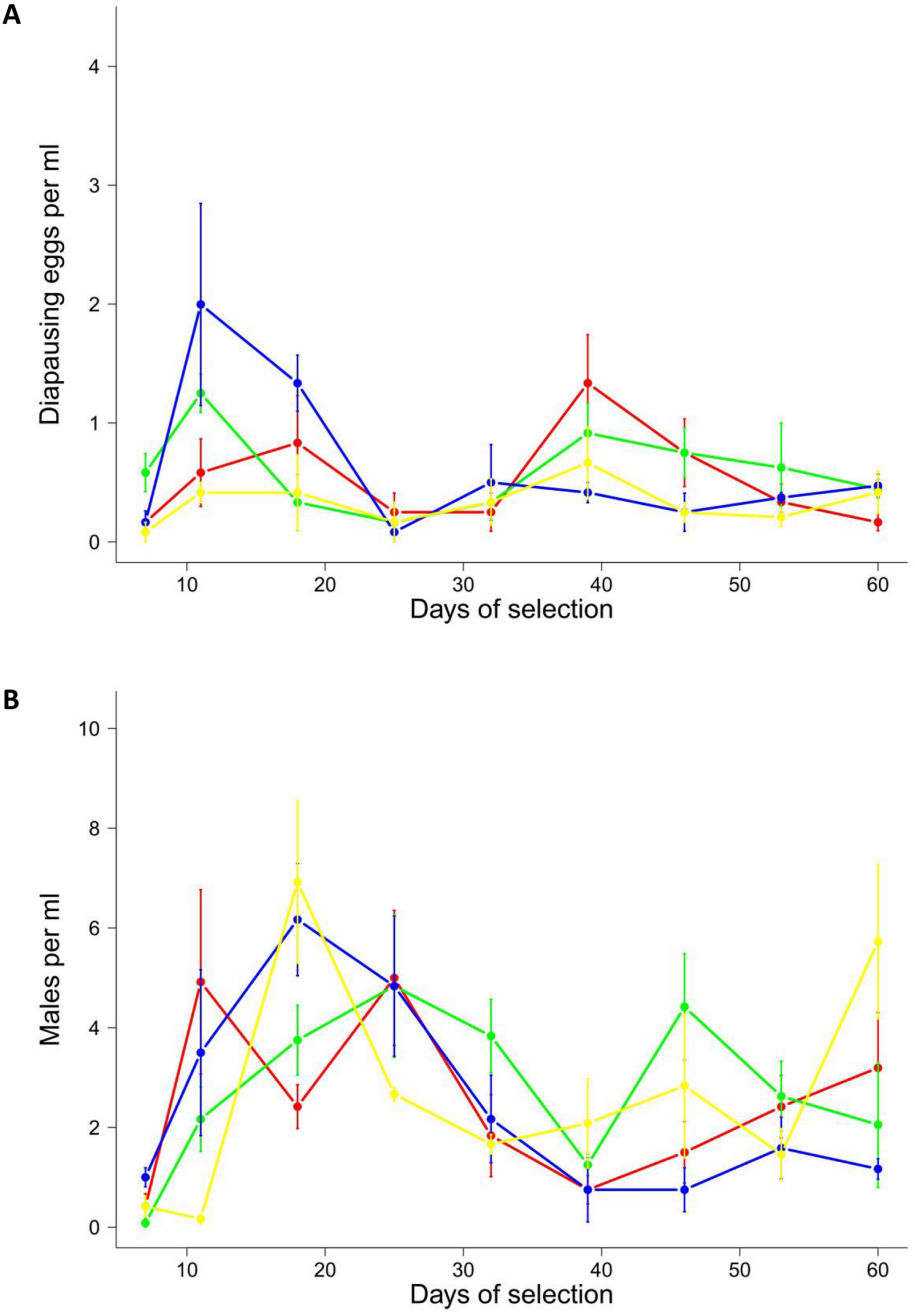
The change of sexual reproduction for *Brachionus calyciflorus* rotifers in the F-S-Environment. (A) Density of diapausing eggs ml^-1^ (dots represent mean values, bars ±1 standard error) and (B) males ml^-1^, both plotted over time. Yellow: A-Populations, Blue: F-Populations, Green: S-Populations and Red: F-S-Populations. Counts are based on three independent samples from each replicate (n = 4).

Since populations might differ in the sexual rates, we also recorded the number of diapausing eggs and males for all populations (Fig 3). Counts of diapausing eggs and males were similar across all treatments, suggesting that the rate of sexual reproduction did not differ among populations (Fig 3).

The initial densities of diapausing eggs for the F- and S-Populations were slightly higher than for A-Populations (Fig 3A, S2 Fig, S2 Table). Diapausing egg density was comparable to the other populations despite a slight decrease observed in the initial phase of the experiment (S2 Fig, S2 Table, Ref.df = 1.9180, F = 3.3710, P = 0.0395). The initial male densities showed no significant differences (Fig 3B). In the A- and F-Populations, the male densities increased initially (S3 Fig, S3 Table, Ref.df = 3.3870, F = 3.13.30, P = 0.0235; Ref.df = 3.7760, F = 8.6010, P<0.0001, respectively). To summarize, since levels of sexual reproduction were comparable between populations, the higher levels of adaptive evolution cannot be explained by differences in the amount of sexual reproduction.

During the last week of the experiment, we measured algae concentrations (optical density) and salt concentrations in all replicates to check the habitat quality and found no difference between the populations. The algae concentrations were stable over the 4 day transfer interval (mean OD 0.0367 ± 0.0075 standard deviation ≅150,000 cells ml^-1^) and were overall comparable across populations, suggesting that both growth and predation were comparable across the populations.

## Discussion

In our experiment the A- and F-S-Populations adaptively evolved faster than the F- or S-Populations (Fig 2), and in the F-S-Populations the main mechanism to initiate adaptive evolution was most likely recombination between beneficial alleles of different loci. This is in agreement with previous studies [10,22], but our results also highlight some important differences. In contrast to our study, a recent publication suggested that the main benefit of sexual recombination consisted in sorting beneficial mutations from deleterious mutations in long-term experimental yeast lines [21]. However, the methodological approach of that study was quite different from ours, as it did not directly measure the initial phenotypic fitness effects of sex, but examined fitness after ~1000 generations and inferred on the mechanism of adaptation by tracking of mutations with whole genome sequencing. It is quite plausible that the role of sex might change during the progress of adaptive evolution: recombination of beneficial mutations might be initially important, whereas mutation clearance might become a dominant factor during later adaptation. Thus, the fitness of the S- and F-Population might have changed through any mechanism if we had run the experiment much longer. The other two mechanisms, liberation of beneficial alleles and mutation clearance, did not seem to play a dominant role in our natural selection experiment. In F- and S-Populations there was a negative linkage disequilibrium after local adaptation; thus sexual recombination should expose deleterious mutations [14], or liberate beneficial alleles from genetic backgrounds of low fitness [11]. Despite the fact that levels of sexual reproduction were high for all populations (Fig 3), those populations could not adapt (Fig 2), and we conclude that both alternative mechanisms were unsuccessful. One potential issue might be that additive genetic variance was too low in the F- and S-Populations [3] and populations might have become monoclonal and homozygous in the initial experiment [24]. This however, is unlikely, considering that in most artificial selection experiments, genetic variation remains high despite strong selection [29]. Furthermore, after soft sweeps, genomic polymorphism remains high in the vicinity of the selected loci because of recombination [30]. The second issue might be that this interpretation only holds for mutation accumulation as basis of local adaptation, while local adaptation could also be caused by antagonistic pleiotropy [25]. We imagine that selection acts on different loci for salt tolerance, rather than on loci for food-limitation, and *vice versa*; thus, mutation accumulation seems more plausible. Further, under pleiotropy, a fitness increase in the F-S-Populations would not be possible. We observed an increase in mean fitness in the F-S-Populations, and the most obvious explanation seems to be that beneficial loci for high-salt and low-food conditions were recombined by sex to match the current selection regime. The deterministic mutation hypothesis (DMH) [13], where deleterious mutations are recombined and removed, is unlikely to explain our findings. If this process would act in F-S-Populations, variation in fitness should decrease, because low fitness variation is removed but no beneficial variation is produced. Not only did the mean fitness increase in the F-S-Populations, but the variance also remained high (S1 Fig), arguing against the DMH. Finally, one potential issue might be frequency dependent selection favouring S-Individuals in the first days and F-Individuals in the remaining days, leaving a higher population density at the end. This is unlikely to be of importance in our experiment considering that the habitat quality remained unchanged over time, thus minimising temporal and/or spatial heterogeneity (bottles were mixed constantly). Further, we added fresh algae every growth period to avoid the evolution of predation-resistant algae clones favouring one sub-population.

The available additive genetic variance potentially increases the observed fitness change despite the low number of generations in contrast to monoclonal designs [14]. We also observed changes in fitness despite the comparably small population size [10] potentially due to the initial -LD. New mutations seem to play a minor role here compared to other studies [10,14], as that would have had an overall effect.

In the A-Populations mean fitness increased almost constantly, and we propose that this response to selection is driven by a mixture of clonal erosion, selection from continuous genetic variation, sexual reproduction and other adaptive mechanisms. Possibly, due to this multitude of mechanisms, adaptive evolution proceeded most rapidly in those populations. This demonstrates the high adaptive potential of these rotifers, as found in other studies [20], and that adaptive evolution may include more mechanisms than just recombination between beneficial alleles of different loci.

The theories regarding sex are advanced and most studies assume selection must act on a sex-modifier shuffling genes [3,4]. We estimated a selection gradient β for the A-Populations via the least squares regression method [31], using fitness measurements relative to the initial fitness at the start of the experiment and found β = ~0.09. This selection gradient is lower than in other studies using artificial selection [12], but would probably be sufficient to favour a modifier of sex, if linked to beneficial alleles of different loci [3,6,18].

In summary, our study shows that sex can speed up adaptation by recombination between beneficial alleles of different loci at the very early stages of adaptive evolution. The importance of other mechanisms (e.g., mutation clearance) might increase during later stages of adaptation, when populations approach their adaptive peak on the fitness landscape. Future studies are needed to elucidate whether there is indeed such a temporal succession of different mechanisms by which sex speeds up adaptation.

## Methods

### Selection experiment

For this experiment we used populations and ancestral clones from our previous experiment [24]. We used this material to assemble the replicates for each population structure. The F-S-Populations consisted of 50% each of F- and S-Individuals (Supporting Information). The A-Populations were established from clonal cultures by adding 20 females of each of the 83 ancestral clones to each replicate. The populations were acclimatized to the constraining conditions by less harsh conditions for the first four days (20 μM nitrogen & 180,000 cells of algae and 2.5 g L^-1^ sea salt). For F-S-Environment we used COMBO medium [32], modified by adding salt (5 g L^-1^) and reduced the available food by limiting nitrogen (10 μM instead of 360 μM), which effectively constrained the growth of the food algae to ~150,000 cells ml^-1^. Populations were grown in 900 ml glass bottles, constantly aerated at 23°C, and continuous illumination was provided with daylight fluorescent bulbs (30–40 μEinstein m^-2^ sec^-1^). Population density was determined twice per week in 3–4 day intervals; the 4-day intervals were analysed. This was done by counting females, diapausing eggs and males under the microscope for three independent samples of each replicate, directly after transfer (T_0_) and at the end of each growth period (T_End_), to estimate growth rates day^-1^. The number of asexual generations was calculated from the average growth rate observed in the experiment (F-S-Populations: growth rate ~0.5 day^-1^) over the 60 day period. We estimated a value of 0.5 sexual generations per week, because diapausing eggs were produced shortly before the transfer to fresh medium (within 3–4 days) and hatched with a delay of three to four days without the dark/cold trigger, which would result in 9–10 sexual generations [24]. However, sexual reproduction is commonly suppressed for the next 5–10 asexual generations in rotifers female hatched from diapausing eggs [33], which reduces the realized number to 3–4 sexual generations.

### Statistical analysis

Statistics were calculated in the R statistical environment [34], using the `mgcv`package [35]. Data were analysed using generalized additive mixed effects modelling (GAMM) with `Population`as a fixed effect. The change of `Fitness`over time `Day`, as continuous variable for each `Population`, was estimated by a smooth term with a *CorExp* correlation structure for `Day`with argument `ID`, where `Replicate`was aggregated with `Population`[36]. We also accounted for different variances of the population by weighting the model, using a *varIdent* heterogeneity structure over `Day`with argument `Population`[36]. Random effects were assumed on the slope `Day`and the argument `ID`. The model was based in the *Poisson* family, used cubic regression, 5 knots and the ´REML´ method. The results of the GAMM remained stable over 3–9 knots, for various correlation structures, and for different corrections of heterogeneity. We calculated various correlation structures and corrections for heterogeneity and selected the best model based on the Akaike`s Information Criterion [36,37]. Model validation was done graphically using diagnostic plots and suggested a high reliability of the presented model. The output of the model provided an intercept, which represents the general level of adaptation in comparison to the A-Populations, and a smooth term, which represents the slope over time as a deviation from zero for each population independently. We calculated the selection gradient **β** from the slope of a linear mixed effect model for the data of the A-Populations separately. Time was treated as continuous variable and the random effect was calculated over `Day`with argument `ID`. This gave us the strength of selection (β) acting in the F-S-Environment on the A-Populations. The variance of fitness was estimated from the mean value and the standard deviation for the sampling periods from days 39–60 divided through the mean of the initial period (days 0–38).

## Supporting information

S1 File Further information on rotifer biology and cultivation methods. (DOCX)

S1 Table Results of the generalized additive mixed effects model on female fitness over time. (DOCX)

S2 Table Results of the GAMM for diapausing eggs over time. (DOCX)

S3 Table Results of the GAMM for males over time. (DOCX)

S1 Fig Estimated relative fitness distribution for the populations during the final experimental period. (DOCX)

S2 Fig The smooth terms over time for densities of diapausing eggs. (DOCX)

S3 Fig The smooth terms over time for densities of males. (DOCX)
