# Supplementary material for: Sex initiates adaptive evolution by recombination between beneficial loci
Source: PLoS One. 2017 Jun 2;12(6):e0177895. doi: 10.1371/journal.pone.0177895 (PMC5456038; doi:10.1371/journal.pone.0177895)
Supplement: S1 File — (DOCX) [file pone.0177895.s001.docx]

**Supporting information**

Sex initiates adaptive evolution by recombination of beneficial loci

**Thomas Scheuerl, Claus-Peter Stelzer**

# Material and Methods

## The study system

As proposed by Fussman (2011), we used rotifers (the Monogonont rotifer *Brachionus calyciflorus*) as model organisms for our evolution experiment, because they can be easily cultured, reproduce quickly and occur at high levels of clonal genetic diversity in nature. Monogonont rotifers are cyclical parthenogens with haploid dwarf males [1], which do not feed any more [2]. Females normally reproduce by ameiotic parthenogenesis, but initiate sporadic sexual episodes, producing sexual and asexual daughters [3]. Induction of sexuality is mainly density dependent [1,4,5]. At high population densities, a chemical cue induces the production of sexual females [5,6]. The oocytes of these sexual females undergo meiosis and develop into haploid males (if not fertilized), or diploid diapausing eggs (if fertilized).

## Stem cultures

Rotifers were initially isolated from Lake `Egelsee`, Austria. Clonal stem cultures of rotifers and algae were maintained at 21°C and continuous illumination was provided with daylight fluorescent bulbs (30–40 µEinstein m^-2^ s^-1^ for rotifers; 200 µEinstein m^-2^ s^-1^ for algae). Clonal cultures of rotifers were re-inoculated twice per week by transferring 6-7 asexually reproducing females to fresh COMBO [7] medium (5 ml) provided in six-well-plates as in previous experiments [8]. Algae were supplied at *ad libitum* concentrations (~400,000 cells ml^-1^).

### Sex and adaptation experiment

The pre-adapted and the genetically variant populations were acclimatized to the constraining conditions by a reduced step for the first four days (20 µMol N & 180.000 cells of algae and 2.5 g L^-1^ oceanic salt). Food algae for the experiment were grown separately in 2 L borosilicate bottles. Algae were concentrated by centrifugation at 3000 rpm for 10 minutes and diluted to the desired food concentration and in the desired medium. At the end of each growth period, the numbers of females, diapausing eggs and males were determined by counting three subsamples fixed with Lugol`s solution using inverted microscopy at 200-fold magnification. The appropriate volume for the next period was filtered using a 30-µm mesh (retaining females, diapausing eggs and partly males) and washed into the newly prepared bottle. All material was rinsed with 70 % alcohol and de-ionized water prior use in the next transfer. After successful transfer, another subsample of living females was counted to receive the initial density T_0_.

**References**

1. Schröder T. Diapause in monogonont rotifers. Hydrobiologia. 2005;546: 291–306. doi:10.1007/s10750-005-4235-x

2. Fussmann GF. Rotifers: excellent subjects for the study of macro- and microevolutionary change. Hydrobiologia. 2011;662: 11–18. doi:10.1007/s10750-010-0515-1

3. Nogrady T, Wallace RL, Snell TW. Rotifera: biology, ecology and systematics. Guides to the identification of the microinvertebrates of the continental waters of the world. Dumont HJF, ed. The Hague (The Netherlands): SPB Academic Publishing. pp 137.; 1993.

4. Gilbert JJ. Specificity of crowding response that induces sexuality in the rotifer Brachionus. Limnol Oceanogr. 2003;48: 1297–1303.

5. Stelzer C-P, Snell TW. Induction of sexual reproduction in Brachionus plicatilis (Monogononta, Rotifera) by a density-dependent chemical cue. Limnol Oceanogr. 2003;48: 939–943.

6. Snell TW, Stelzer C-P. Removal of surface glycoproteins and transfer among Brachionus species. Hydrobiologia. 2005;546: 267–274. doi:10.1007/s10750-005-4207-1

7. Kilham S, Kreeger D, Lynn S, Goulden C, Herrera L. COMBO: a defined freshwater culture medium for algae and zooplankton. Hydrobiologia. 1998;377: 147–159. doi:10.1023/A:1003231628456

8. Scheuerl T, Riss S, Stelzer C-P. Phenotypic effects of an allele causing obligate parthenogenesis in a rotifer. J Hered. 2011;102: 409–415. doi:10.1093/jhered/esr036

9. Kondrashov AS. Deleterious mutations and the evolution of sexual reproduction. Nature. 1988;336: 435–440. doi:10.1038/336435a0

10. Scheuerl T, Stelzer C-P. Patterns and dynamics of rapid local adaptation and sex in varying habitat types in rotifers. Ecol Evol. 2013;3: 4253–4264. doi:10.1002/ece3.781

Raw Data
