# Supplementary material for: Sex initiates adaptive evolution by recombination between beneficial loci
Source: PLoS One. 2017 Jun 2;12(6):e0177895. doi: 10.1371/journal.pone.0177895 (PMC5456038; doi:10.1371/journal.pone.0177895)

**S1 Table. Results of the generalized additive mixed effects model on female fitness over time.** The parametric coefficients give the intercept of the model and show that all pre-adapted populations had a higher initial fitness (Fig 2). The smooth terms calculated by the model to describe the change in fitness over time deviated only in the A-Populations and in the F-S-Populations proposing an increase in fitness over time (Fig 2). The model was calculated with a *CorExp* correlation structure, *varIdent* correction for heterogeneity, cubic regression and 5 knots.


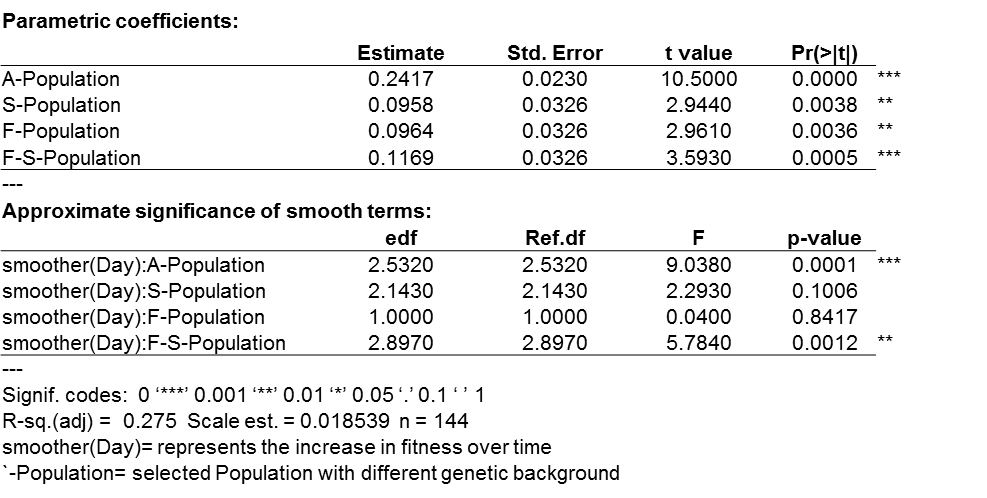

Supplement: S1 Table — (DOCX) [file pone.0177895.s002.docx]
