# Supplementary material for: Sex initiates adaptive evolution by recombination between beneficial loci
Source: PLoS One. 2017 Jun 2;12(6):e0177895. doi: 10.1371/journal.pone.0177895 (PMC5456038; doi:10.1371/journal.pone.0177895)
Supplement: S3 Table — (DOCX) [file pone.0177895.s004.docx]

**S3 Table. Results of the GAMM for males over time.** The model was calculated for different heterogeneities corrected by a *varExp* structure over time for the different genetic population structures (Fig 3B). The other model parameters were the same as before.
