# Supplementary material for: Sex initiates adaptive evolution by recombination between beneficial loci
Source: PLoS One. 2017 Jun 2;12(6):e0177895. doi: 10.1371/journal.pone.0177895 (PMC5456038; doi:10.1371/journal.pone.0177895)

**S1 Fig. Estimated relative fitness distribution for the populations during the final experimental period.** The normal distribution curves are estimated from the mean value and the standard deviation for the sampling periods from days 39-60 divided through the mean of the initial period (days 0-38). The curves represent potential fitness distribution within the populations relative to fitness of the initial period indicated by 1 for the “ancestor” populations. Assuming the deterministic mutation hypothesis [9] for the F-S-Populations, the mean should increase as selection would remove the low fitness variation. However, the standard deviation in fitness would decrease, as there would be limited positive variation. The red line would fall on all other lines on the right side of the curves. A less contaminated genome, pushing the mean of the red line forward, would rely on replacing deleterious mutations either with neutral variation from its own subpopulation (high variation due to not previously selected [10]), or with loci from the alternative subpopulation, which is the recombination of beneficial loci (low variation because previously selected). As the standard deviation in fitness was actually higher for F-S-Populations than F- or S-Populations, represented by the area under the red curve on the right side, this strongly points into the direction that beneficial loci were recombined and genotypes of higher fitness were produced. The A-Populations massively increased in fitness and in variance in fitness. Yellow: A-Populations, Blue: F-Populations, Green: S-Populations and Red: F-S-Populations.


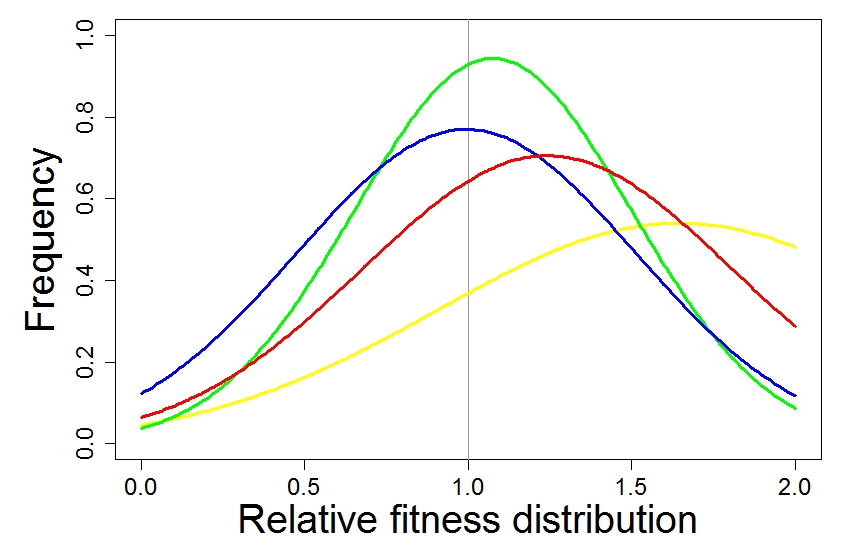

Supplement: S1 Fig — (DOCX) [file pone.0177895.s005.docx]
