# Supplementary material for: Sex initiates adaptive evolution by recombination between beneficial loci
Source: PLoS One. 2017 Jun 2;12(6):e0177895. doi: 10.1371/journal.pone.0177895 (PMC5456038; doi:10.1371/journal.pone.0177895)

**S3 Fig. The smooth terms over time for densities of males.** The GAMM was calculated for different heterogeneities corrected by a *varExp* structure over time for the different genetic population structures. The other model parameters were the same as before. Significances are provided in S3 Table.


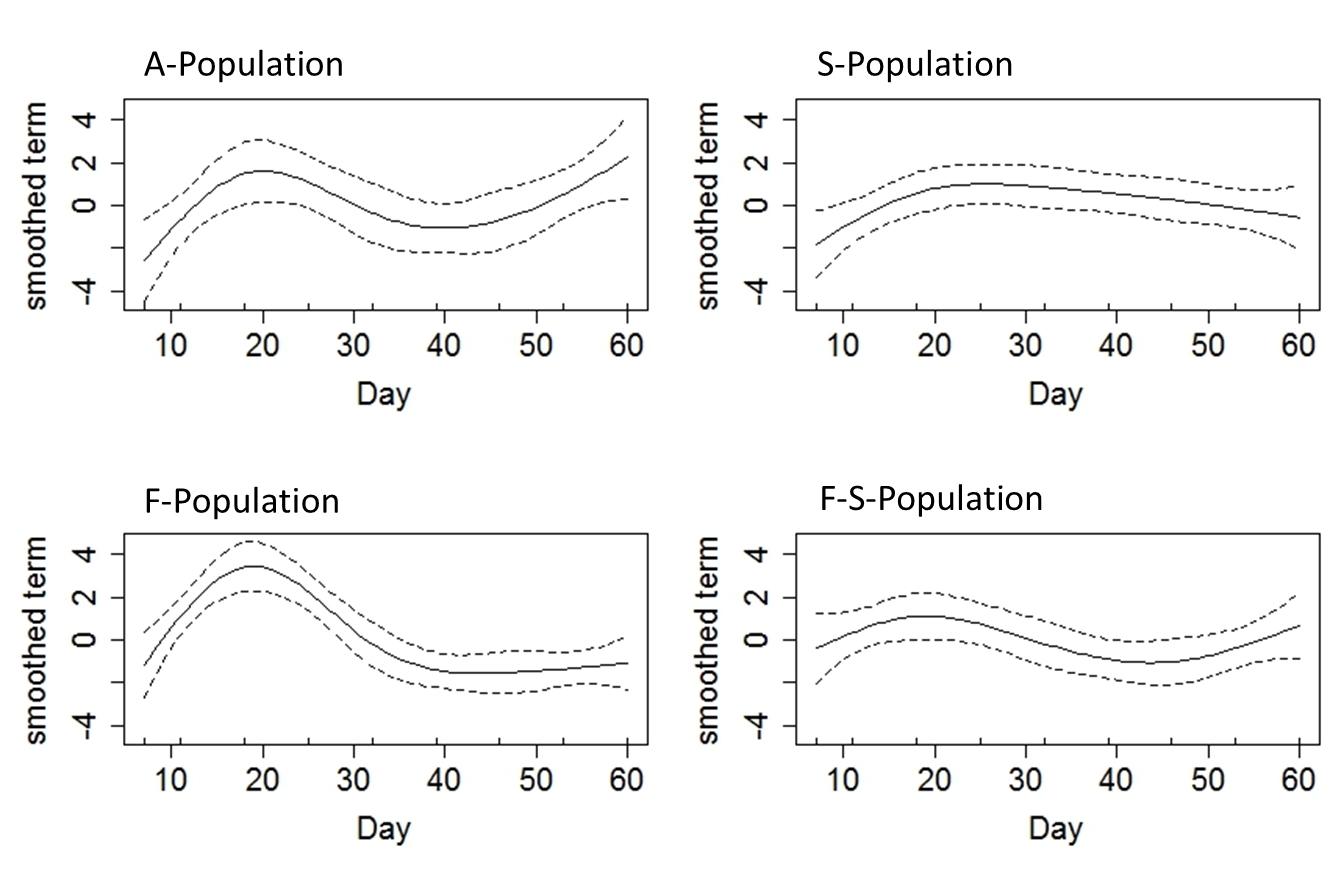

Supplement: S3 Fig — (DOCX) [file pone.0177895.s007.docx]
